# Supplementary material for: Fat-Soluble Vitamers: Parent-Child Concordance and Population Epidemiology in the Longitudinal Study of Australian Children
Source: Nutrients. 2022 Nov 24;14(23):4990. doi: 10.3390/nu14234990 (PMC9735774; doi:10.3390/nu14234990)
Supplement: Supplementary file 1 [file nutrients-14-04990-s001.zip › nutrients-1894585-supplementary.pdf]

Supplementary Materials:

Table S1: Concentrations (ng/mL) of the standard curve.

Compound	Std 1	Std 2	Std 3	Std 4	Std 5	Std 6	Std 7
retinol	1600	800	400	200	100	50	25
retinoic acid	115	57.5	28.75	14.38	7.19	3.59	1.80
25-OH-D_3	364.35	182.18	91.09	45.54	22.77	11.39	5.69
1-α-25$(OH)_2$-D_3	165	82.5	41.25	20.63	10.31	5.16	2.58
α-tocopherol	14000	7000	3500	1750	875	437.5	218.75
γ-tocopherol	2000	1000	500	250	125	62.5	31.25
α-tocotrienol	100	50	25	12.5	6.25	3.13	1.56
K1	20	10	5	2.5	1.25	0.63	0.31
MK-4	20	10	5	2.5	1.25	0.63	0.31
MK-7	30	15	7.5	3.75	1.88	0.94	0.47

Abbreviations: Std: Standards and Std1: Original Stock concentrations

Table S2: External standard concentrations spiked into heparin plasma to make QC's.

Compound	QC 1	QC 2	QC 3
retinol	1600	400	100
retinoic acid	115	28.75	7.19
25-OH-D_3	364.35	91.09	22.77
1-α-25$(OH)_2$-D_3	165	41.25	10.31
α-tocopherol	14000	3500	875
γ-tocopherol	2000	500	125
α-tocotrienol	100	25	6.25
K1	20	5	1.25
MK-4	20	5	1.25
MK-7	30	7.5	1.88

Abbreviations: QC: Quality Control

Table S3: Mixed model results of sex and generation effects on FSV concentrations. The sex effect was on adults only, not on children.

	Vitamer	Condition	Estimated change ng/mL	p-value
Vitamin A	retinol	Effect of sex	-0.004	0.75
		Effect of generation	0.26	<0.001
		Interaction between Sex vs Generation	0.19	<0.001
	retinoic acid	Effect of sex	-0.01	0.66
		Effect of generation	0.17	<0.001
		Interaction between Sex vs Generation	0.15	<0.001
Vitamin D	25-OH-D_3	Effect of sex	0.07	<0.01
		Effect of generation	-0.1	<0.001
		Interaction between Sex vs Generation	-0.008	0.82
Vitamin E	α-tocopherol	Effect of sex	-0.02	0.16
		Effect of generation	0.2	<0.001
		Interaction between Sex vs Generation	-0.06	0.02
	γ-tocopherol	Effect of sex	-0.08	0.02
		Effect of generation	0.18	<0.001
		Interaction between Sex vs Generation	-0.02	0.77
	α-tocotrienol	Effect of sex	0.04	0.4
		Effect of generation	-0.07	0.05
		Interaction between Sex vs Generation	0.004	0.96

Table S4: Published reference plasma/serum concentrations of fat-soluble vitamers

Vitamin	Vitamer	Sample characteristics	Concentrations	Reference
A	retinol	Middle-aged Chinese women aged 32-75 y (n=404)	1.22 ± 0.34 µmol/L (252.08 – 446.87 ng/mL)<br>1.56 ± 0.38 µmol/L (338.02 – 555.73 ng/mL)	25
		Child controls (n=14)	59 ± 5.88 µg/dL (531.2 – 644.8 ng/mL)<br>Range 28.7-119 µg/dL (287-1190 ng/mL)	26
	retinoic acid	Non-diabetic subjects (55.7 ± 9.5 y), men (n=511) and women (n=314)	Men 1.92 (1.32–2.49) ng/mL<br>Women 1.99 (1.38–2.55) ng/mL	27
		Healthy women (n = 36; age 19–47 y)	9.3 ± 3.7 nmol/L (1.68 – 3.90 ng/mL)	28
D	25-OH-D_3	Group of school children (n=479) aged 5-12 y	Males 75.9 ± 21 nmol/L (21.9 – 38.82 ng/mL)<br>Females 70.8 ± 18.3 nmol/L (21.03 - 35.7 ng/mL)	29
		Control subjects (n=208) males and females, age y 25.6±0.5	Males 93.9±2.7 nmol/L (36.54– 38.70 ng/mL)<br>Females 99.7±2.9 nmol/L (38.78 – 41.10 ng/mL)	30
		Control subjects aged 68.7 ± 7.2 y Caucasian (n = 110)	114.21 ± 50.6 nmol/L (25.48 - 66.02 ng/mL)	31
E	α-tocopherol	Healthy children (n=166); 1 month - 18 years	11.9 - 30 µmol/L (5,125 – 12,921 ng/mL)	32
		Healthy 20 – 59 y old adults (males (n=33) and females(n=73))	Males 15.45 ± 10.16 µmol/L (2278.45 - 11,030 ng/mL)<br>Females 15.00 ± 4.54 µmol/L (4,505.22 – 8,416 ng/mL)	33
		Control subjects aged 68.7 ± 7.2 y Caucasian (n = 110)	19.18 ± 8.85 µg/mL (10330- 29030 ng/mL)	31
	γ-tocopherol	Control subjects aged 68.7 ± 7.2 y Caucasian (n = 110)	1.67 ± 1.48 µg/mL (190 – 3150 ng/mL)	31
		Control subjects aged 68.7 ± 7.2 y Mean values (n=675)	1.98 ± 1.36 µg/mL (620 – 3340 ng/mL)	
		Male and Female participants aged 56.3 y	0.04 to 0.61 mg/dL (400- 6100 ng/mL)	34
	α-tocotrienol	Males (n=36) age 21 - 30 y	34.3 ± 9.6 ng/mL	35
		Males after postprandial diet (n=10)	(1.46 ± 0.52 µmol/L) 399.18 – 840.82 ng/mL	36
		Healthy male adults aged (n=64) age 20–26 y	9.9 ± 2.5 ng/ml	37
		Placebo male adults aged (n=16) age 20–26 y	10.31± 3.70 ng/ml	

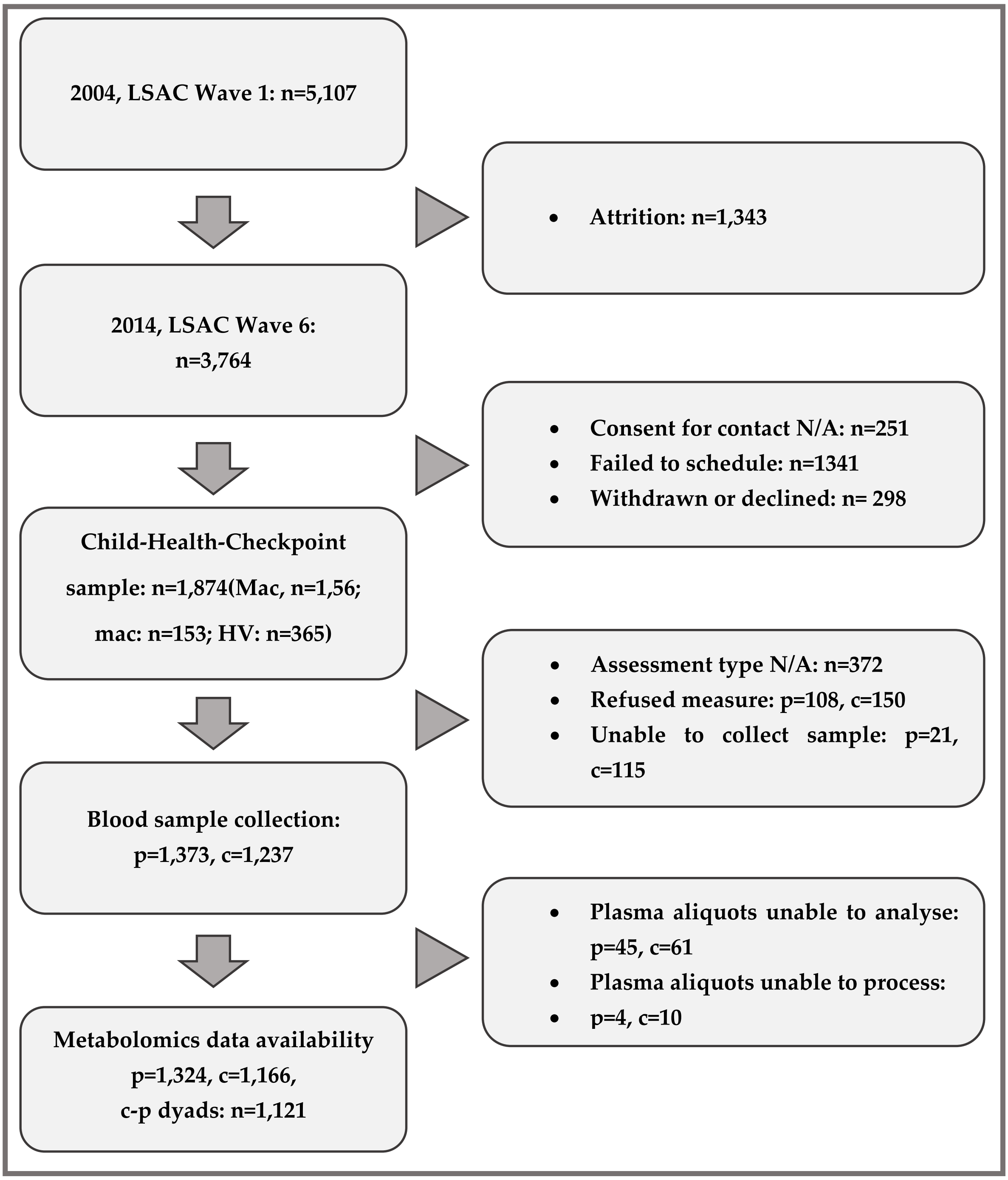

Figure S1: Participant information chart. HV: home visit; LSAC: Longitudinal Study of Australian Children; Mac: main assessment centre; mac: mini assessment centre; p: parent adults; c: children.

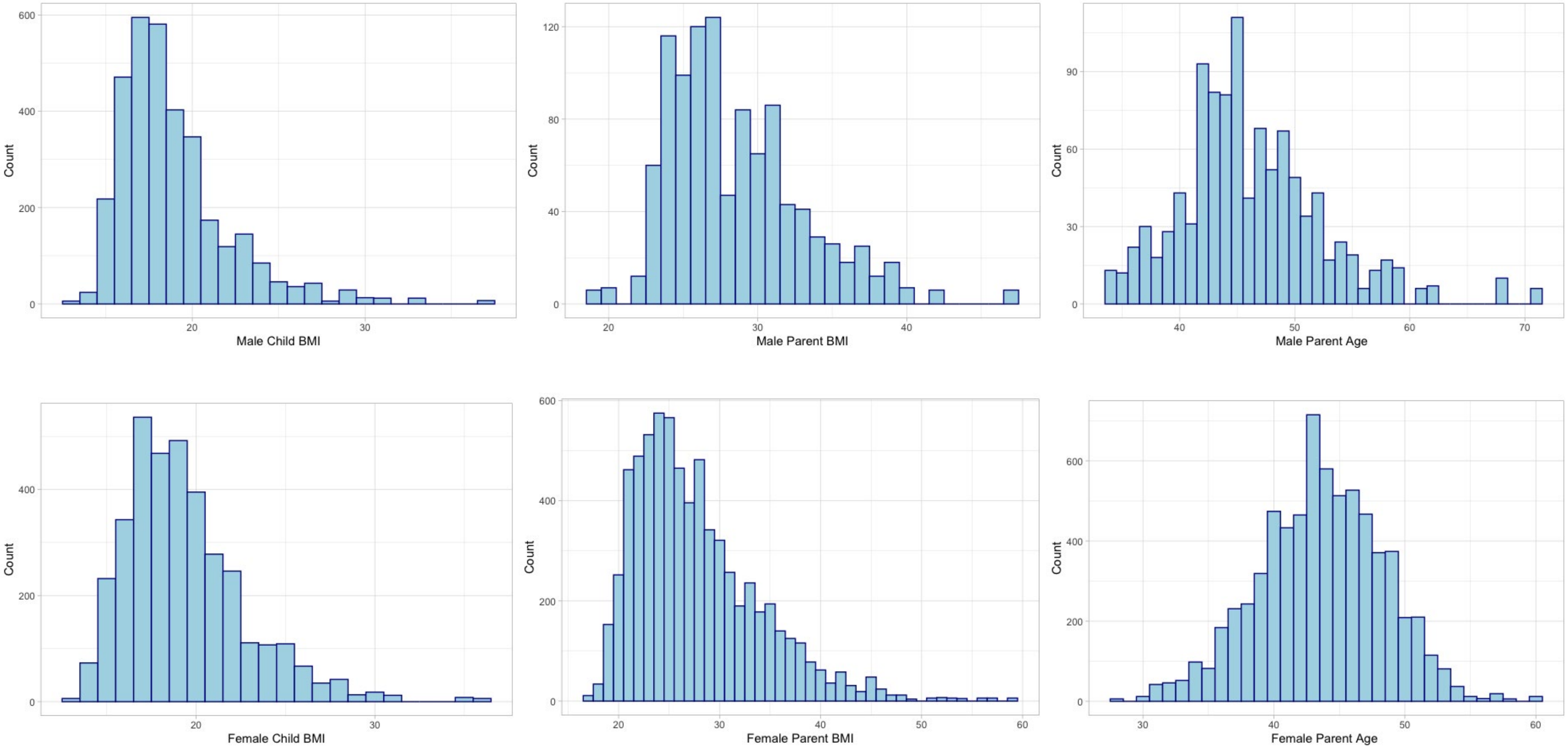

Figure S2: Population characteristics of the LSAC's Checkpoint cohort.

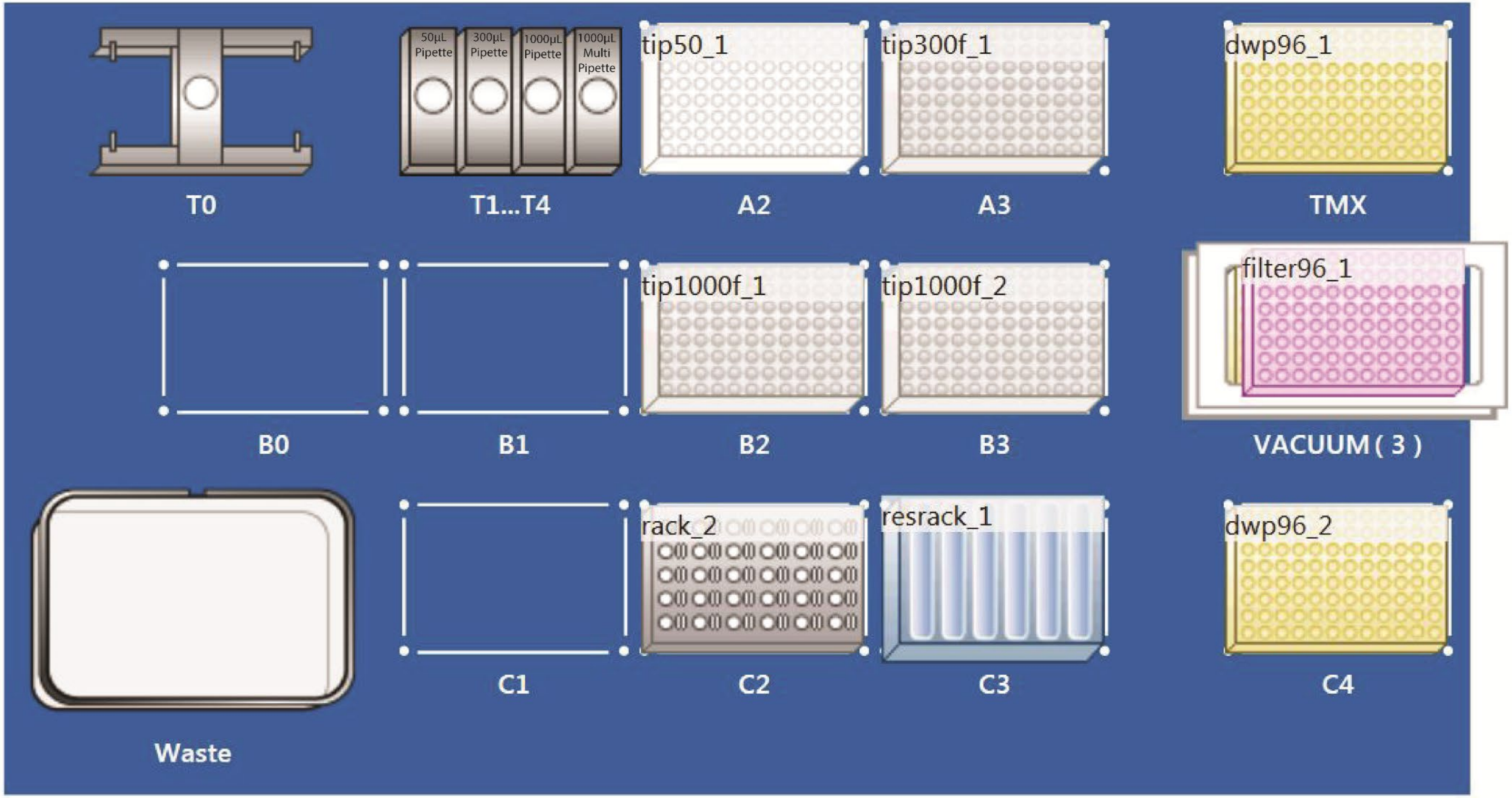

Figure S3: The Eppendorf EpMotion liquid handling robot setup. T0; Plate mover, T1-T4; Automated pipettor, A2, A3, B2 and B3; Filtered pipette tips, dwp96: 96 Deep well plates, Vacuum; vacuum manifold, resrack; reservoirs (3mL and 10mL), rack2; 2mL 2 ml Eppendorf tube holder.

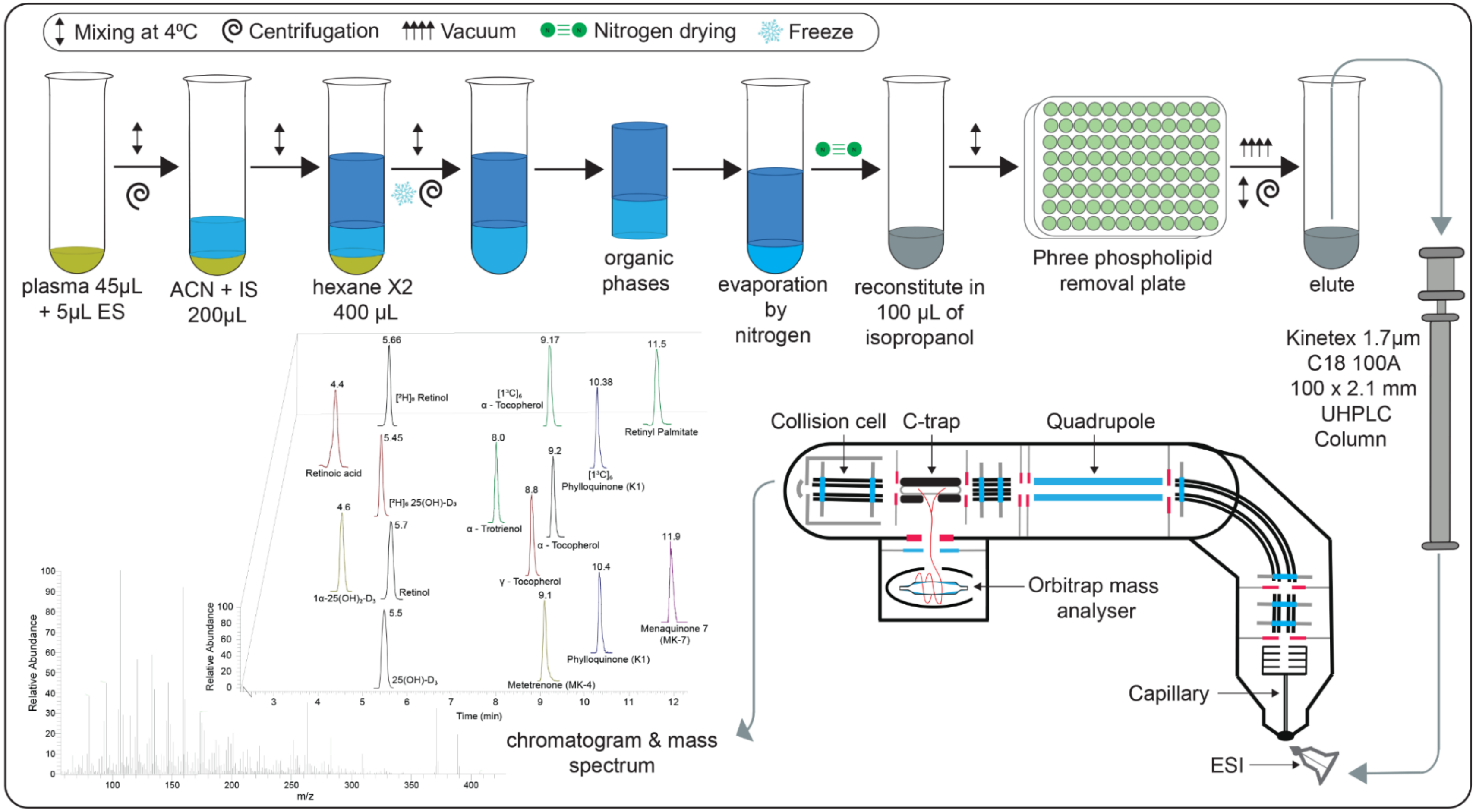

Figure S4: Workflow for sample preparation and LC-MS/MS analysis (Adopted from Arachchige et al., 2021; attribution 4.0 International (CC BY 4.0)).
